# Characteristics of Dye-Sensitized Solar Cells with TiO_2_ Stripes

**DOI:** 10.3390/ma15124212

**Published:** 2022-06-14

**Authors:** Wen-Feng Lai, Pei-Ling Chao, Xin-Yu Lin, Yin-Pei Chen, Jih-Hsin Liu, Tz-Feng Lin, Wei-Chou Hsu, Chia-Yi Huang

**Affiliations:** 1 Institute of Microelectronics, National Cheng Kung University, Tainan 701, Taiwan; q18031027@gs.ncku.edu.tw; 2 Department of Applied Physics, Tunghai University, Taichung 407, Taiwan; s08210016@thu.edu.tw (P.-L.C.); s07210046@thu.edu.tw (X.-Y.L.); s07210044@thu.edu.tw (Y.-P.C.); 3 Department of Electrical Engineering, Tunghai University, Taichung 407, Taiwan; jhliu64@thu.edu.tw; 4 Department of Fiber and Composite Materials, Feng Chia University, Taichung 407, Taiwan; tflin@fcu.edu.tw; 5 Academy of Innovative Semiconductor and Sustainable Manufacturing, National Cheng Kung University, Tainan 701, Taiwan

**Keywords:** dye-sensitized solar cell, TiO_2_ stripe, energy conversion efficiency, surface area, electron transport path

## Abstract

A TiO_2_ strip array with a thickness of 90 nm was fabricated by photolithography and physical vapor deposition. This work utilized the chemical and physical methods to fabricate the TiO_2_ strip array. A porous semiconductor layer made of TiO_2_ nanoparticles was coated on the TiO_2_ strip array. The TiO_2_ strip array has a one-dimensional protrusive structure. The energy conversion efficiency (4.38%) of a dye-sensitized solar cell (DSSC) with the TiO_2_ strip array exceeded that (3.20%) of a DSSC without a TiO_2_ strip array by 37%. In addition, this result was verified by the electrochemical impedance spectra of the two DSSCs. Therefore, the TiO_2_ strip array can be used to increase the energy conversion efficiencies of DSSCs. The large energy conversion efficiency of the DSSC with the TiO_2_ strip array arises from the large surface area of the one-dimensional protrusive structure and its specific electron transport paths. The DSSC with the TiO_2_ strip array has advantages of economical production cost, easy fabrication, and boosting energy conversion efficiency.

## 1. Introduction

Dye-sensitized solar cells (DSSCs) have attracted much attention due to their flexibilities, simple structures, and low fabrication costs [1,2,3,4,5,6,7,8,9,10]. DSSCs, perovskite solar cells, and silicon-based solar cells have achieved energy conversion efficiencies of 13.0%, 25.7%, and 27.6%, respectively, nowadays. DSSCs have smaller energy conversion efficiencies than other types of solar cells. This drawback hinders the practice applications of DSSCs [1,2,3,4,5,6,7,8,9,10]. As a result, the improvement of energy conversion efficiencies of DSSCs is a critical need for achieving next-generation solar cells. 

A normal DSSC has a structure of substrate/transparent conductive oxide (TCO) layer/porous semiconductor layer with dye molecules/electrolyte layer/counterelectrode layer/substrate. As the dye molecules absorb solar energy, they generate electrons and holes. The electrons are injected into the conduction band of the porous semiconductor layer because the lowest unoccupied molecular orbital (LUMO) of the dye has a higher energy level than the conduction band. The electrons are transported from the porous semiconductor layer to an external circuit via the TCO layer because the porous semiconductor layer has a higher conduction band than the TCO layer. The transportation causes the loss of the electrons of the dye molecules, so the dye molecules are oxidized. The electrolyte molecules cause the reduction of the oxidized dye molecules, so the electrolyte molecules regenerate the dye molecules. The electrolyte molecules lose their electrons due to the regeneration of the dye molecules, so the electrolyte molecules are oxidized. The electrons from the external circuit cause the reduction of the oxidized electrolyte molecules, so the electrons regenerate the electrolyte molecules. The electrons are recycled in the DSSC and external circuit, so the DSSC generates electrical energy. 

A conventional porous semiconductor layer is made of TiO_2_ nanoparticles. The TiO_2_ nanoparticles have large surface areas, so a large number of dye molecules are adsorbed to their surfaces. The dye molecules that absorb solar energy can generate many electrons. However, the nanoparticles are a zero-dimensional structure [11]. Because the electrons in the zero-dimensional structure have no specific transporting paths, they are easy to recombine with holes. The recombination of the electrons and holes reduces the energy conversion efficiency of a DSSC. A good porous semiconductor layer allows electrons to be transported in specific paths, increasing the energy conversion efficiency of a DSSC. Pavasupree et al. used a porous semiconductor layer that is made of TiO_2_ nanoparticles and TiO_2_ nanorods to fabricate a DSSC [12]. The energy conversion efficiency of the DSSC is larger than that of a DSSC with a porous semiconductor layer that is made of only TiO_2_ nanoparticles by 22%. This result reveals that the TiO_2_ nanorods allow electrons to be transported in specific paths, increasing the energy conversion efficiency of the DSSC. Asagoe et al. utilized a porous semiconductor layer that is made of 90% TiO_2_ nanoparticles and 10% TiO_2_ nanowires to fabricate a DSSC [13]. The energy conversion efficiency of the DSSC is larger than that of a DSSC with a porous semiconductor layer that is made of 100% TiO_2_ nanoparticles by 17%. This result reveals that the TiO_2_ nanowires with a concentration of 10% in the TiO_2_ nanoparticles facilitate the electron transport in the DSSC. However, the energy conversion efficiency of the DSSC decreased significantly as the concentration of the TiO_2_ nanowires in the TiO_2_ nanoparticles increased from 10% to 100%. This result arises from the fact that the TiO_2_ nanowires with a concentration that exceeds 10% in the TiO_2_ nanoparticles form the entangled structure that obstructs the electron transport in the DSSC. Therefore, TiO_2_ nanostructures that are fabricated only by chemical synthesis cannot further increase the energy conversion efficiencies of DSSCs. 

Choi et al. reported that a counterelectrode with a patterned TiO_2_ layer enhances the photon harvesting in a DSSC due to the light reflection from the patterned TiO_2_ layer [14]. Baba et al. reported that a patterned substrate that is coated with a gold film increases the energy conversion efficiency of a DSSC due to the light scattering from the grating substrate [15]. It is interesting to use a patterned photoelectrode to increase the energy conversion efficiency of a DSSC. 

A TiO_2_ strip array with a thickness of 90 nm was fabricated using photolithography and physical vapor deposition in this work. The TiO_2_ strip array exhibited a one-dimensional protrusive structure. The protrusive structure increased the surface area of the TiO_2_ strip array, and offered the specific paths for electron transport. Therefore, the energy conversion efficiency of a DSSC with the TiO_2_ strip array was larger than that of a DSSC without a TiO_2_ strip array by 37%. The TiO_2_ strip array has potential in developing next-generation solar cells. This work proposed the chemical and physical methods for increasing the energy conversion efficiencies of DSSCs using TiO_2_ strip arrays. The methods have many advantages such as various TiO_2_ structures, economical production cost, and easy fabrication.

## 2. Materials and Methods

**Figure 1:**
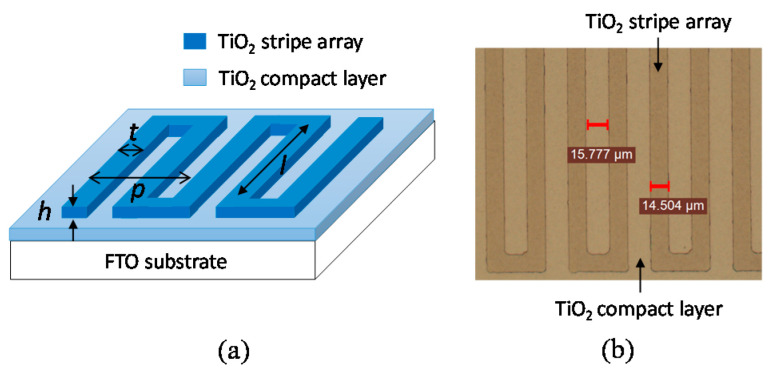
(**a**) Design and (**b**) optical microscope image of the TiO_2_ stripe array.

Figure 1a presents the design of a TiO_2_ stripe array that is deposited on a TiO_2_ compact layer. Each of the TiO_2_ stripes in the array is designed to have height (*h*), linewidth (*t*), period (*p*), and length (*l*) of 90 nm, 15 μm, 60 μm, and 5 mm, respectively. The TiO_2_ stripe array was created by photolithography and physical vapor deposition. A TiO_2_ compact layer with a thickness of 22 nm was deposited on a glass substrate with a fluorine-doped tin oxide (FTO) layer (Ruilong, Miaoli County, Taiwan) using a magnetron sputter. The FTO layer functions as a TCO layer in this work. The TiO_2_ compact layer in a DSSC was used to not only prevent the contact of dye molecules and a TCO layer but also facilitate the contact of a porous semiconductor layer and the TCO layer [16,17]. An ENPI202 photoresist (Everlight Chemical Industrial, Tainan, Taiwan) was coated on the TiO_2_ compact layer using a spin coater. The time and speed of the spin were 40 s and 4500 rpm for the coating of the photoresist, respectively. Stripe patterns in a photomask were transferred onto the ENPI photoresist layer by the irradiation of UV light. The ENPI photoresist was developed using its developer. The photoresist stripe patterns were obtained following the development. TiO_2_ was deposited on the photoresist stripe patterns using the magnetron sputter. The FTO glass was immersed into acetone to remove the photoresist stripe patterns. The TiO_2_ stripe array was obtained following the immersion. Figure 1b presents the optical microscope image of the TiO_2_ stripe array. Therefore, the TiO_2_ stripe array was reliable in this work. 

**Figure 2:**
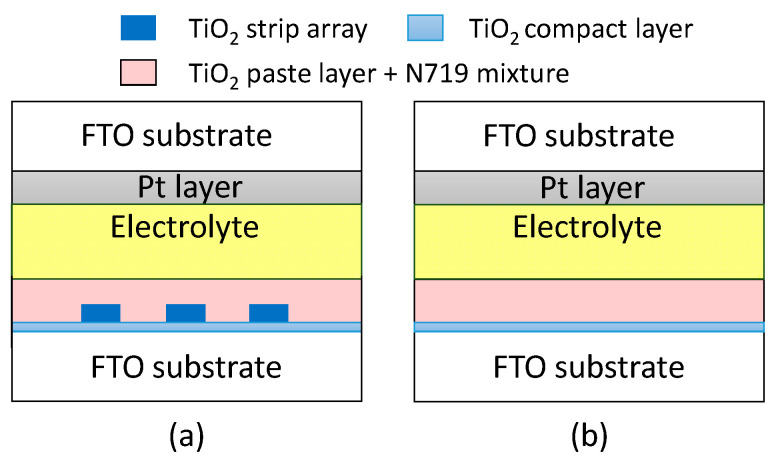
Schematic configuration of DSSCs (**a**) with and (**b**) without TiO_2_ strip array.

Figure 2a,b present the schematic configuration of DSSCs with and without a TiO_2_ strip array, respectively. A TiO_2_ paste layer made of TiO_2_ nanoparticles was coated on the TiO_2_ stripe array using a blade coater. The TiO_2_ paste layer functioned as a porous semiconductor layer in this work. The TiO_2_ paste layer was heated from room temperature to 500 °C with a step of 5 °C/min, and held at a temperature of 500 °C for 45 min. The temperature of the TiO_2_ paste layer was decreased from 500 °C to room temperature using natural cooling. The TiO_2_ paste layer was immersed into a N719 mixture of 50 mL acetonitrile, 50 mL tert-Butanol, and 59.4 mg D719 dye (Everlight Chemical Industrial, Tainan, Taiwan) for 24 h. The TiO_2_ compact layer, TiO_2_ stripe array, TiO_2_ paste layer, and N719 mixture with a concentration of 0.5 mM compose a photoelectrode in this work. A Pt layer with a thickness of 50 nm was deposited on a FTO glass using the magnetron sputter. The FTO glass with the Pt layer functioned as a counterelectrode in this work. An empty cell was obtained by separating the photoelectrode and counterelectrode using a Surlyn film with a thickness of 20 μm. The empty cell was filled with an EL-200 electrolyte (Everlight Chemical Industrial, Tainan, Taiwan). The DSSC with the TiO_2_ stripe array was obtained following the filling of the electrolyte. Figure 2b displays that a DSSC has the same geometrical structure as the design of Figure 2a, but has no TiO_2_ stripe array. The DSSC without a TiO_2_ stripe array was used to evaluate the energy conversion efficiency of the DSSC with the TiO_2_ stripe array. 

All the experiments in this work were carried out using twenty DSSCs with TiO_2_ strip arrays and twenty DSSCs without TiO_2_ strip arrays. The experimental data in this work are the averages of the measured values of the DSSCs with and without the TiO_2_ strip arrays.

## 3. Results and Discussion

**Figure 3:**
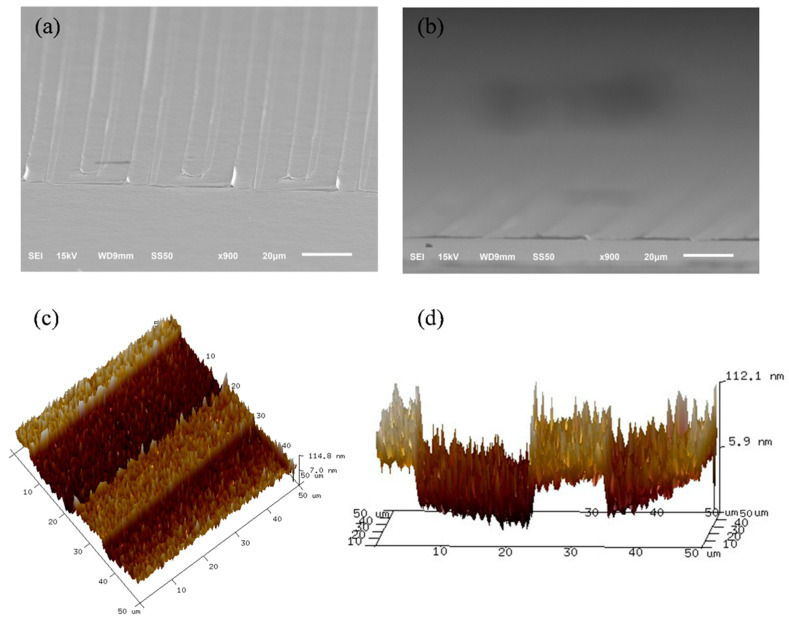
Scanning electron microscope images of the TiO_2_ stripe array that is deposited on TiO_2_ compact layer at (**a**) 45° and (**b**) 80° angles of incidence, respectively. (**c**) Top and (**d**) side views of atomic force microscope images of the TiO_2_ strip array that is deposited on TiO_2_ compact layer.

Figure 3a,b present the scanning electron microscope images of the TiO_2_ stripe array that was deposited on the TiO_2_ compact layer at 45° and 80° angles of incidence, respectively. The TiO_2_ stripe array and its design have similar geometrical dimensions. The TiO_2_ stripe array was successfully fabricated by the photolithography and physical vapor deposition. The TiO_2_ stripe array has a one-dimensional protrusive structure, so the layer has a large surface area and specific paths for electron transport. Figure 3c,d present the top and side views of the atomic force microscope (AFM) images of the TiO_2_ strip array that was deposited on the TiO_2_ compact layer, respectively. The AFM images provide reliable data on the actual relief of the surface of the TiO_2_ strip array. 

**Figure 4:**
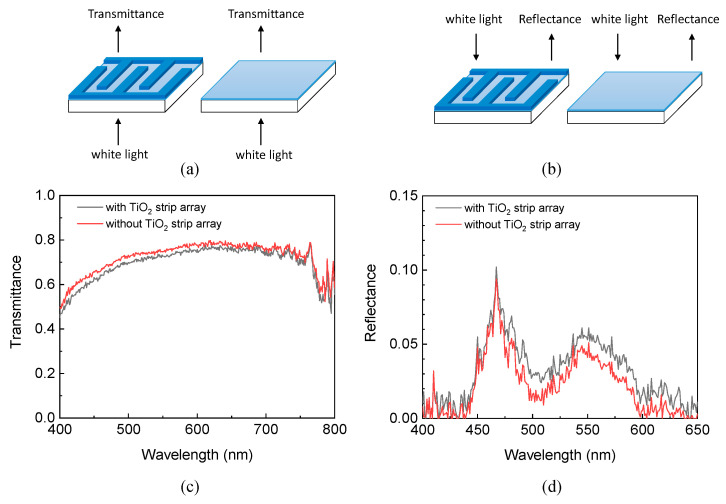
Setups of (**a**) transmission and (**b**) reflection spectra of FTO glass substrates with and without the TiO_2_ strip array. (**c**) Transmission and (**d**) reflection spectra of FTO glass substrates with and without the TiO_2_ strip array.

Figure 4a,b displays the setup of the experimental transmission (reflection) spectra of the FTO glass substrates with and without the TiO_2_ strip array. The spectra were measured using a transmission and reflection measurement system (LSRT-R, LiveStrong Optoelectronics, Kaohsiung, Taiwan). The system includes an optical fiber, white-light source, photometric integrating sphere, and spectrometer. The wavelength of the system ranges from 300 nm to 900 nm, and its spectral resolution is 1 nm. The optical fiber was connected with the white-light source, and guided the light from the source. The light was incident from the side of the FTO glass substrates (compact TiO_2_ layers) for measuring the transmission (reflection) spectra of the FTO glass substrates with and without the TiO_2_ strip array. The transmissions (reflections) of the FTO glass substrates with and without the TiO_2_ strip array were normalized with that of air (a white broad). 

Figure 4c presents that the FTO glass substrate with the TiO_2_ strip array had a lower transmittance at a wavelength than that without a TiO_2_ strip array because the TiO_2_ strip array scattered the light that was incident from the side of the FTO glass substrate. This result implies that the TiO_2_ strip array may hinder incident photons from going into a DSSC, so the DSSC has a small energy conversion efficiency. Figure 4d displays that the FTO glass substrate with the TiO_2_ strip array had a larger reflectance at a wavelength than that without a TiO_2_ strip array because the TiO_2_ strip array scattered the light that was incident from the side of the compact TiO_2_ layer. This result implies that the TiO_2_ strip array may confine the photons that are reflected by the Pt layer of a DSSC in its TiO_2_ compact layer, so the DSSC has a large energy conversion efficiency. The results in Figure 4c,d reveal that the TiO_2_ strip array caused the competition between the entrance of the incident photons and the confinement of the reflected photons. Therefore, it is interesting to study the effect of a TiO_2_ strip array to the energy conversion efficiency of a DSSC. 

**Figure 5:**
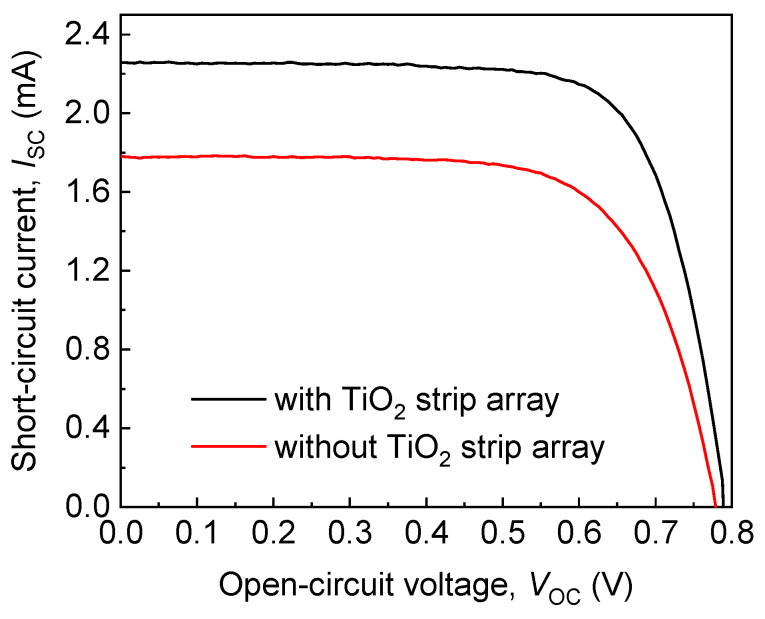
Dependences of short-circuit currents (*I*_SC_) of DSSCs with and without TiO_2_ strip array on open-circuit voltages (*V*_OC_).

Figure 5 displays the dependences of the short-circuit currents (*I*_SC_) of the DSSCs with and without the TiO_2_ strip array on their open-circuit voltages (*V*_OC_). The *I*_SC_-*V*_OC_ curves in Figure 5 were obtained using a source meter (2400, Keithley, Beaverton, OR, USA) and AM 1.5 solar simulator (XES-40S2, San-EI Electric, Osaka, Japan). The DSSCs with and without the TiO_2_ stripe array had an identical active area of 5 mm × 6 mm in this work.

The averaged photovoltaic parameters of the DSSCs with and without the TiO_2_ strip array are summarized in Table 1. The DSSC with the TiO_2_ strip array had a larger open-circuit voltage, short-circuit current, and fill factor than that without a TiO_2_ strip array. This result reveals that the energy conversion efficiency of the DSSC with the TiO_2_ strip array was larger than that of the DSSC without a TiO_2_ strip array by 37%. The large energy conversion efficiency of the DSSC with the TiO_2_ strip array arises from the one-dimensional protrusive structure of the TiO_2_ strip array and specific electron transport paths of the structure. The DSSCs with and without the TiO_2_ strip array had low energy conversion efficiencies because the thickness of the TiO_2_ paste layers in the DSSCs was not optimized. Efforts are being made at the authors’ laboratory to optimize the thickness of the TiO_2_ paste layer in a DSSC with a TiO_2_ strip array, the results of which will be published in the near future.

This work studied the effect of photoelectrodes with TiO_2_ strip arrays on the energy conversion efficiencies of DSSCs. The DSSC cell comprising the photoelectrode with the TiO_2_ strip array was 37% higher than that comprising the photoelectrode without a TiO_2_ strip array because the TiO_2_ strip array in the former facilitates the electron transport. Choi et al. studied the effect of counterelectrodes with TiO_2_ strip arrays on the energy conversion efficiencies of DSSCs [14]. The DSSC cell comprising the counterelectrode with the TiO_2_ strip array had a 22% higher energy conversion efficiency than that comprising the counterelectrode without a TiO_2_ strip array because the TiO_2_ strip array in the former enhanced the photon harvesting. The results in our and Choi’s works revealing that the improvement of photoelectrodes and counterelectrodes can increase the energy conversion efficiencies of DSSCs.

**Figure 6:**
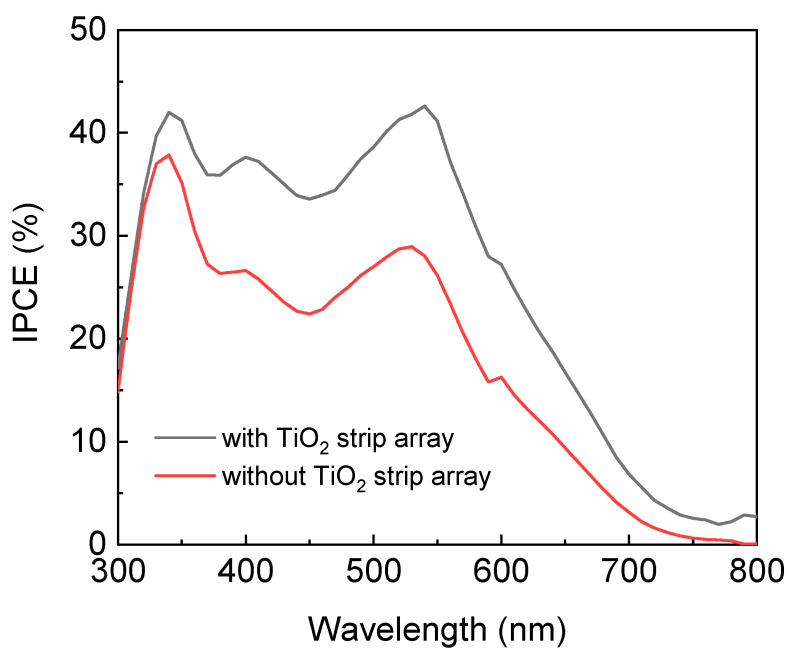
IPCE spectra of DSSCs with and without a TiO_2_ strip array.

The incident photon-to-current efficiency (IPCE) spectra of the DSSCs with and without the TiO_2_ strip array were measured using a measurement system (LSQE-N, LiveStrong Optoelectronics, Kaohsiung, Taiwan). Figure 6 presents the IPCE spectra of the DSSCs with and without the TiO_2_ strip array. The DSSC with the TiO_2_ strip array had larger IPCEs at the frequencies of its spectrum than that without a TiO_2_ strip array. This result verifies the DSSC with the TiO_2_ strip array had a larger short-circuit current than that without a TiO_2_ strip array due to $J_{\mathrm{SC}}=q\int \varphi (\lambda )\times IPCE(\lambda )\times d\lambda$, where *J*_SC_ is the short-circuit current density of a DSSC; *q* is the charge of an electron; *φ*(*λ*) is the wavelength-dependent photon fluxes of an incident light; and *IPCE*(*λ*) is the wavelength-dependent IPCEs of the DSSC.

**Figure 7:**
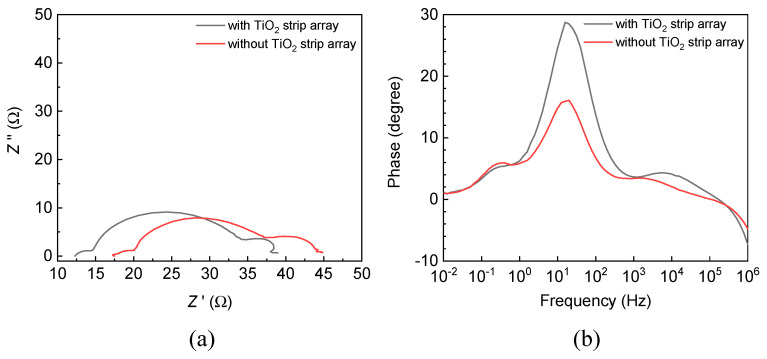
(**a**) Nyquist and (**b**) Bode plots of DSSCs with and without a TiO_2_ strip array.

The electrochemical impedance spectra of the DSSCs with and without the TiO_2_ strip array were measured using a potentiostat/galvanostat (WonATech, ZIVE SP1, Seoul, Korea) at a solar intensity of 100 mW/cm^2^ and bias voltage of 10 mV, and the frequency in the measurement ranged from 10 mHz to 1 MHz. Figure 7a,b present the Nyquist and Bode plots of the DSSCs with and without TiO_2_ strip array. The plots were obtained from the electrochemical impedance spectra of the DSSCs with and without the TiO_2_ strip array. 

Table 2 displays the averaged series resistances (*R*_s_, *R*_ct_, and *R*_ec_), averaged peak frequencies (*f*_p_) of the Bode plots, and averaged electron lifetimes (*τ*) for the DSSCs with and without the TiO_2_ strip array. *R*_s_ is a series resistance related to the electron transport in the FTO/photoelectrode interface; *R*_ct_ is a series resistance related to the electron transport in the electrolyte/Pt layer interface; and *R*_rec_ is a series resistance related to the electron transport in the photoelectrode/electrolyte interface. *f*_p_ is a peak frequency of the second semi-circle of a DSSC in its Bode plot. *τ* is given by *τ* = 1/(2π × *f*_p_). 

The DSSC with the TiO_2_ strip array had a smaller *R*_s_ than the DSSC without a TiO_2_ strip array. This result reveals that the electron transport from the DSSC to an external circuit was more efficient in the DSSC with the TiO_2_ strip array than in the DSSC without a TiO_2_ strip array. The DSSC with the TiO_2_ strip array had a larger *R*_rec_ than that without a TiO_2_ strip array. This result displays that the electron injection from the LUMO of the dye to the conduction band of the photoelectrode was more efficient in the DSSC with the TiO_2_ strip array than in that without a TiO_2_ strip array. The DSSC with the TiO_2_ strip array had a larger *τ* than that without a TiO_2_ strip array. This result reveals that the recombination of the electrons and holes had a smaller probability in the DSSC with the TiO_2_ strip array than in that without a TiO_2_ strip array. The small *R*_s_, large *R*_rec_, and large *τ* of the DSSC with the TiO_2_ strip array reveal that the TiO_2_ strip array increased the energy conversion efficiency of the DSSC because its one-dimensional protrusive structure has a large surface area and specific electron transport paths. The DSSCs with and without the TiO_2_ strip array had similar *R*_ct_ due to their identical electrolyte and Pt layer.

## 4. Conclusions

This work developed a design through photolithography and physical vapor deposition to fabricate the TiO_2_ strip array with a thickness of 90 nm. The TiO_2_ paste layer made of the TiO_2_ nanoparticles was coated on the TiO_2_ strip array. The TiO_2_ strip array had a one-dimensional protrusive structure. The protrusive structure increased the surface area of the TiO_2_ strip array, and offered specific paths for the electron transport in the DSSC with the TiO_2_ strip array. Therefore, the energy conversion efficiency of the DSSC with the TiO_2_ strip array was larger than that of the DSSC without a TiO_2_ strip array by 37%. The TiO_2_ strip array has potential in developing next-generation solar cells. This work utilized chemical and physical methods to fabricate the TiO_2_ strip array. The methods have advantages of various TiO_2_ structures, low production cost, and easy fabrication.

## Figures and Tables

**Table 1:** Averaged photovoltaic parameters of DSSCs with and without the TiO_2_ strip array.

DSSC	*V*_OC_ (mV)	*I*_SC_ (mA)	FF (%)	Efficiency (%)
with strip array	788 ± 0	2.26 ± 0.05	0.74 ± 0.01	4.38 ± 0.09
without strip array	775 ± 0	1.78 ± 0.06	0.70 ± 0.01	3.20 ± 0.13

**Table 2:** Averaged series resistances (*R*_s_, *R*_ct_, and *R*_ec_), averaged peak frequencies (*f*_p_) of Bode plots, and averaged electron lifetimes (*τ*) for DSSCs with and without a TiO_2_ strip array.

DSSC	*R*_s_ (Ω)	*R*_ct_ (Ω)	*R*_rec_ (Ω)	*f*_p_ (Hz)	*τ* (ms)
with strip array	12.3 ± 0.1	2.1 ± 0.14	20.1 ± 0.52	15.9 ± 0.05	10.0 ± 0.05
without strip array	17.5 ± 0.1	2.6 ± 0.11	17.1 ± 0.25	20.0 ± 0.05	7.9 ± 0.05

## Data Availability

Data is contained within the article.
